# Kidney Transplantation in a Patient With Hairy Cell Leukemia in Complete Remission: A Case Report

**DOI:** 10.1002/jha2.70424

**Published:** 2026-11-13

**Authors:** Andrea Poli De Frias, Carla Romagnoli, Francesca Polit, Fernando Poli De Frias, Leily Santos, Alexandra Lyubimova, Vathany Sriganeshan, Jacqueline C. Barrientos

**Affiliations:** ^1^ Department of Internal Medicine Mount Sinai Medical Center Miami Beach Florida USA; ^2^ Mount Sinai Braman Comprehensive Cancer Center Miami Beach Florida USA; ^3^ Department of Pathology Mount Sinai Medical Center Miami Beach Florida USA; ^4^ Mount Sinai Medical Center Miami Beach Florida USA

**Keywords:** case report, hairy cell leukemia, immunosuppression, kidney transplantation, vemurafenib

## Abstract

Hairy cell leukemia (HCL) is a rare, indolent B‐cell neoplasm characterized by the BRAF V600E mutation. Kidney transplantation in patients with active or prior hematologic malignancy represents a complex clinical scenario with limited evidence on current guidelines. We report the first documented case of a patient that while undergoing kidney transplant evaluation was diagnosed with classic HCL. He was treated with vemurafenib and rituximab and achieved complete remission (CR). He subsequently underwent successful kidney transplantation and has remained in sustained CR with stable allograft function at 22 months of follow‐up. This case highlights the importance of individualized multidisciplinary transplant decision‐making.

**Trial Registration**: The authors have confirmed clinical trial registration is not needed for this submission.

## 1 Introduction

Hairy cell leukemia (HCL) is a rare, indolent B‐cell lymphoproliferative disorder that often achieves durable remissions with appropriate treatment [1]. The annual incidence is approximately 0.3 cases per 100,000 individuals, resulting in 1000–1300 new diagnoses each year in the United States. In patients with end‐stage renal disease (ESRD), kidney transplantation offers a superior survival and quality‐of‐life over long‐term dialysis [2]. Kidney Disease: Improving Global Outcomes (KDIGO) guidelines generally exclude candidates with active malignancy from transplantation due to concerns for recurrence under immunosuppression, with exceptions for select indolent cancers [2, 3, 4]. Evidence guiding transplant eligibility in patients with hematologic malignancies, specifically HCL, remains limited.

## 2 Case Presentation

A 72‐year‐old man with ESRD was undergoing evaluation for kidney transplantation when he was found to have symptomatic anemia refractory to erythropoietin therapy and red blood cell transfusions. Laboratory evaluation revealed macrocytic anemia (Table 1), and a bone marrow (BM) biopsy confirmed the diagnosis of classic HCL (cHCL) (Figures 1 and 2; Table 2).

**TABLE 1:** Laboratory data at presentation and after treatment.

Parameter	At presentation	After treatment
White blood cell count (WBC)	5.04 × 10^3^/µL	6.38 × 10^3^/µL
Hemoglobin (Hgb)	6.8 g/dL	10.5 g/dL
Mean corpuscular volume (MCV)	100.5 fL	104.1 fL
Platelet count	148 × 10^3^/µL	126 × 10^3^/µL
Absolute neutrophil count (ANC)	3.58 × 10^3^/µL	4.20 × 10^3^/µL

**FIGURE 1:**
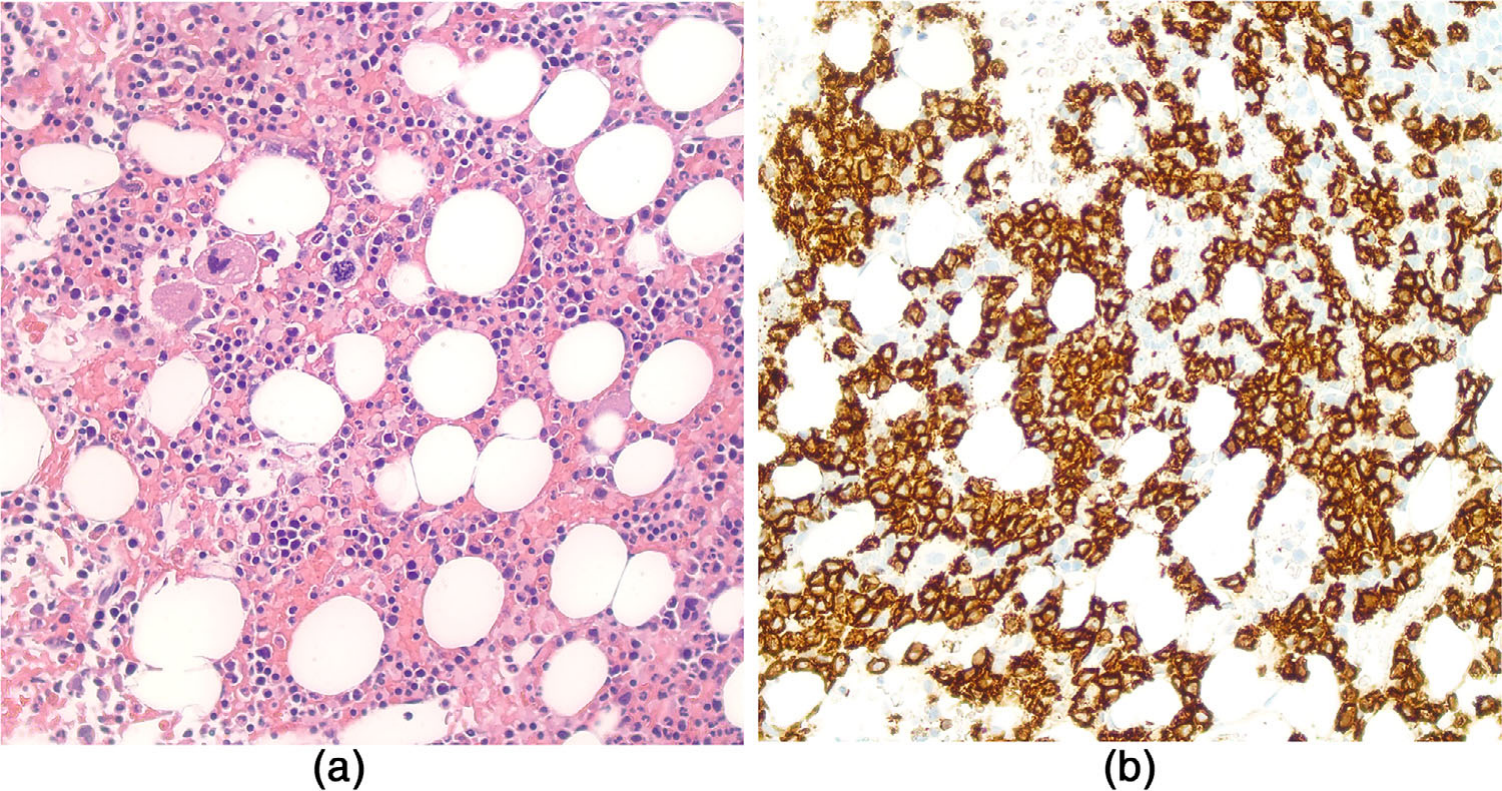
BM biopsy (a) hematoxylin–eosin stain showing subtle HCL. In this example, the process is highlighted by focally increased cellularity; (b) diffuse, subtle interstitial involvement by hairy cell leukemia is most readily seen with CD20 staining, as shown in this bone marrow core biopsy.

**FIGURE 2:**
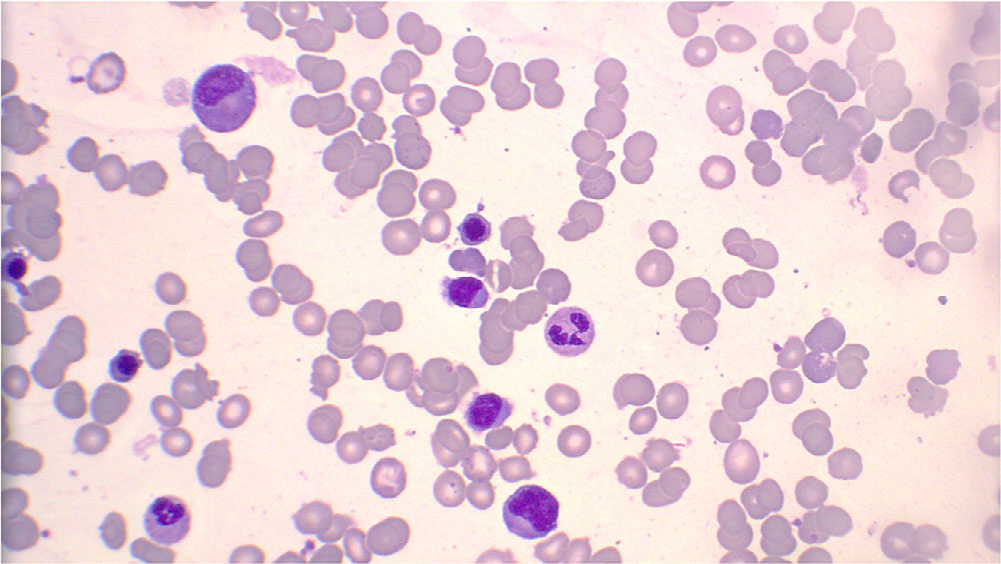
BM aspirate showing atypical lymphoid cells with basophilic cytoplasm and circumferential “hairy” projections, along with round to oval nuclei displaying finely dispersed, spongy chromatin.

**TABLE 2:** Bone marrow biopsy findings at diagnosis and after treatment.

Feature	Biopsy 1—at diagnosis (August 2022)	Biopsy 2—posttreatment (June 2023)
Histopathology	Normocellular marrow for age with trilineage hematopoiesis and adequate iron stores. Involvement by low‐grade B‐cell lymphoma comprising 20%–30% of marrow cellularity.	Normocellular marrow for age with trilineage hematopoiesis and increased iron stores. No morphologic or immunophenotypic evidence of residual hairy cell leukemia.
Bone marrow aspirate	Normocellular particles; adequate megakaryocytes; normal erythroid; and granulocytic maturation. Lymphocytes with small mature morphology, some exhibiting cytoplasmic projections.	Normocellular particles; adequate megakaryocytes with no morphologic abnormalities; normal erythroid and granulocytic maturation.
Flow cytometry	Small 5.1% monoclonal B‐cell population detected with positive expression for CD19, CD20, CD200, CD11c, CD103, and kappa light chain; negative for CD5, CD25, and CD10—immunophenotype consistent with hairy cell leukemia. T‐cells 14.7% (CD4/CD8 ratio 2.1:1); NK cells 1.7%; plasma cells 0.5%. No discrete blast population.	No immunophenotypic abnormalities detected. B‐cells 0.03% (polyclonal); T‐cells 9% (CD4/CD8 ratio 1.7:1); NK cells 1.3%; plasma cells 0.2%; blasts 0.9%, no aberrant immunophenotype.
Karyotype	46, XY [20]—normal male karyotype.	N/A
Immunohistochemical	Annexin‐1 positive; BCL1 negative. CD20 and PAX‐5 highlight increased B‐cells occupying 20%–30% of marrow cellularity. CD3 positive in T‐cells. CD34 positive without increase in immature cells. CD71 positive in erythroid precursors; CD61 positive in megakaryocytes.	N/A
Molecular assay	*BRAF* V600E mutation detected (c.1799T>A). *MYD88* mutation: negative.	N/A

Kidney transplantation was deferred because of the newly diagnosed active hematologic malignancy. Although cladribine remains the standard first‐line therapy for cHCL, it was not considered an optimal treatment option because of concerns regarding increased toxicity associated with impaired renal clearance in the setting of ESRD. Furthermore, the potential for prolonged immunosuppression, with an increased risk of serious infections, was considered undesirable in a patient anticipated to undergo kidney transplantation shortly.

Following discussion with the International HCL consortium, the consensus recommendation was to initiate vemurafenib at a reduced dose with subsequent dose escalation as tolerated, in combination with rituximab. Early during treatment, the patient developed a non‐pruritic cutaneous rash, abdominal discomfort, hyperuricemia, and hyperkalemia, the latter requiring a brief interruption of vemurafenib. Therapy was subsequently resumed. This occurred concurrently with progressive deterioration of renal function, ultimately necessitating the initiation of hemodialysis. Rituximab was initiated as planned, and the patient successfully completed treatment with full dose vemurafenib.

After completing therapy, repeat BM biopsy demonstrated no morphologic or immunophenotypic evidence of residual HCL (Figure 3; Table 2). Hematologic parameters also improved across all cell lineages (Table 1), consistent with a complete remission (CR).

**FIGURE 3:**
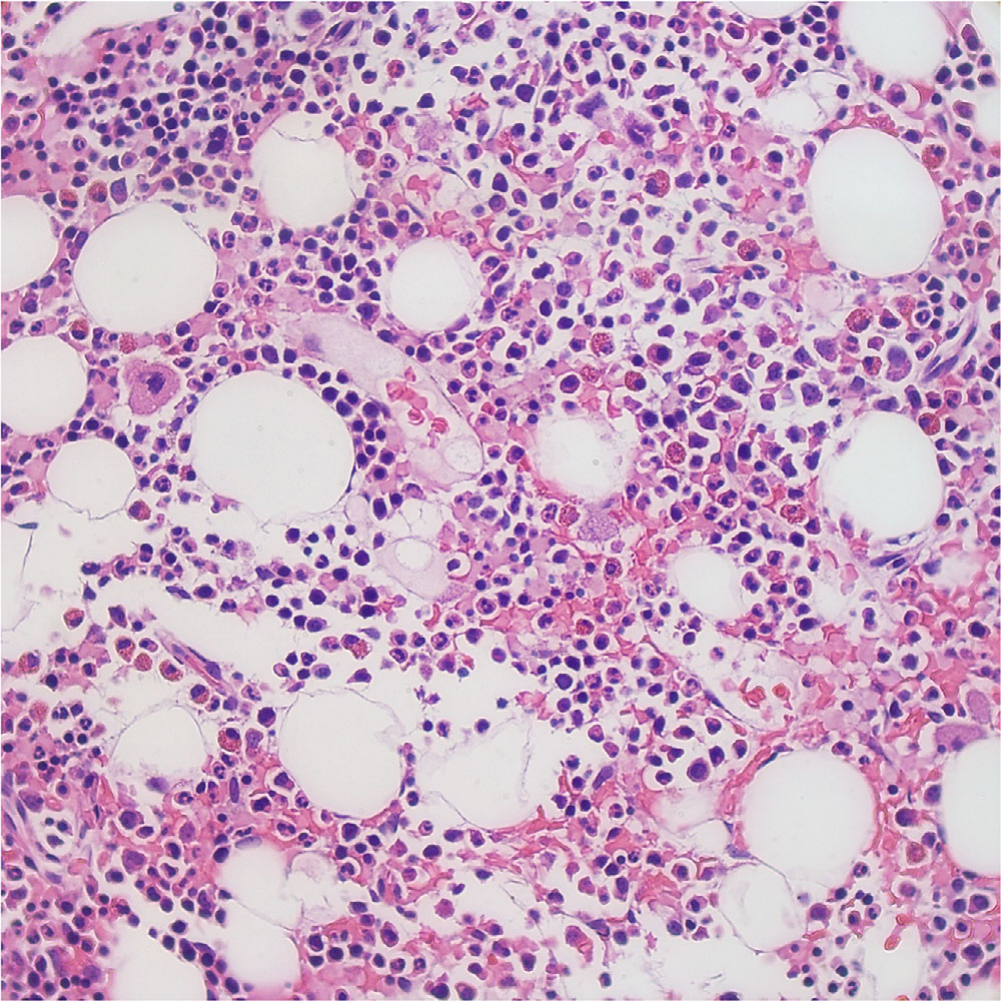
Normocellular bone marrow appropriate for age, demonstrating trilineage hematopoiesis with normal maturation, with no evidence of involvement by hairy cell leukemia.

Before transplantation, the patient had been evaluated by multiple transplant centers across different cities, all of which declined to proceed because of the recent diagnosis of HCL and recommended a minimum disease‐free interval of 5 years before reconsidering transplant eligibility. Given the patient's age, such a delay would likely have precluded kidney transplantation altogether. In addition, the increased mortality associated with long‐term maintenance hemodialysis further emphasized the need for timely transplantation once CR had been achieved.

Following multidisciplinary discussion, the patient was deemed eligible to proceed with kidney transplantation without a mandated disease‐free waiting period. He remained hemodialysis‐dependent until he underwent living‐donor kidney transplantation 17 months after achieving CR. Induction immunosuppression consisted of methylprednisolone and thymoglobulin, followed by maintenance therapy with extended‐release tacrolimus and mycophenolate mofetil. The patient experienced a respiratory syncytial virus (RSV) infection prior to transplantation, but no other infections occurred at any point during treatment or follow‐up. At the most recent follow‐up, 22 months after transplantation, the patient remained in CR with stable allograft function and no need for hemodialysis.

## 3 Discussion

HCL comprises < 2% of leukemias and is characterized by BM and splenic infiltration by lymphocytes with characteristic cytoplasmic projections, frequently causing pancytopenia. The *BRAF* V600E mutation, present in most patients with cHCL, has improved diagnostic accuracy and enabled targeted therapy [1, 5].

Treatment is indicated for symptomatic cytopenias, recurrent infections, or one or more of the following: Absolute neutrophil count < 1000/µL, hemoglobin < 11 g/dL, or platelet count < 100.000/µL [1, 2, 3, 4, 5]. Purine nucleoside analogs (PNAs; cladribine or pentostastin) remain first‐line therapy, achieving high CR rates and long‐term survival [1, 5, 6]. For patients who are unfit for PNA therapy or who have refractory disease, *BRAF* inhibitor‐based regimens such as vemurafenib or dabrafenib have been incorporated into National Comprehensive Cancer Network (NCCN) guidelines despite their off‐label use. Combined with rituximab, these agents can produce rapid hematologic responses, high rates of minimal residual disease (MRD)‐negative CR, and durable remissions with limited myelotoxicity [4, 7, 8, 9, 10, 11].

In our patient, treatment selection was particularly challenging because of ESRD, advanced age, and the anticipated need for kidney transplantation requiring prolonged posttransplant immunosuppression. PNAs were considered suboptimal because impaired renal clearance in ESRD may increase the risk of drug toxicity. In addition, the well‐recognized myelotoxicity and prolonged immunosuppression associated with PNAs, which can predispose patients to serious infections, were undesirable in a patient expected to undergo solid organ transplantation [1, 5, 6, 7].

Following achievement of CR, the HCL Consortium supported proceeding with kidney transplantation, considering that the anticipated survival and quality‐of‐life benefits outweighed the potential risks associated with transplantation after treatment for HCL [2, 3, 4, 12]. However, transplant candidacy in patients with a history of malignancy remains challenging due to concerns regarding recurrence risk under chronic immunosuppression [3, 4, 12]. Current KDIGO guidelines exclude candidates with active malignancy but offer no specific waiting periods or eligibility criteria for indolent or low‐grade hematologic malignancies, instead recommending joint hematology‐transplant evaluation given the risk of accelerated progression under immunosuppression (not graded). However, HCL is not specifically addressed in these guidelines. While such policies are often based on observational studies demonstrating increased risks of mortality, graft loss, and malignancy in transplant recipients with prior cancer, these data are heterogeneous and frequently derived from solid tumors or aggressive hematologic malignancies and may not be directly applicable to HCL [3, 4]. HCL‐specific relapse posttransplant data is limited to a single inconclusive case report after cardiac transplant [13]. Given the biology of HCL and the availability of sensitive MRD markers, confirmed MRD‐negative CR could be a more scientifically grounded eligibility criterion than arbitrary time‐based periods [3, 4]. In this case, transplantation was delayed 17 months despite confirmed CR, due to center‐specific requirements for fixed cancer‐free waiting periods.

## 4 Conclusion

This case demonstrates the use of vemurafenib plus rituximab as a bridge to kidney transplantation in a patient with cHCL who remains in CR 22 months after transplantation despite ongoing immunosuppression. These findings support individualized transplant eligibility based on depth of remission rather than fixed waiting periods and add to the limited evidence guiding the management of patients with HCL requiring solid organ transplantation. They also underscore the importance of patient education and shared decision‐making to achieve better‐individualized outcomes.

## Author Contributions

Jacqueline Barrientos, Alexandra Lyubimova, and Leily Santos‐Carrion were involved in patient care and collected clinical information. Francesca Polit and Vathany Sriganeshan did pathological analysis. Andrea Poli De Frias, Fernando Poli De Frias, Carla Romagnoli, and Jacqueline Barrientos wrote the manuscript. All authors critically reviewed and approved the final manuscript.

## Funding

The authors have nothing to report.

## Ethics Statement

The authors have nothing to report.

## Consent

Consent was obtained from the patient for publication of this case report and any accompanying images.

## Conflicts of Interest

Jaqueline Barrientos has received research support from BeOne, Janssen, and Abbvie; consulting fees from BeOne, AstraZeneca, Janssen, and BMS; and has participated on the Data Safety Monitoring Board or Advisory Board of BeOne, AstraZeneca, Janssen, and BMS. The other authors declare no conflicts of interest.

## Patient Perspective

What began as a routine examination by my physician ended up as a “you need to check this out” moment. A biopsy and pet scan led to a diagnosis of hairy cell lymphoma by one oncologist and the transfer to another with specific knowledge and expertise in the disease and its potential cure. My physician, in particular, and her staff, clearly put me on the road to recovery. Frequent blood tests, monitoring, a couple of transfusions, infusions and medication ordered by my oncologist led me on the correct path to beating the illness. And even if an unacceptable result occurred, it did not alter her instilling confidence and encouragement in meeting a successful end. No doubt, her unbelievable ability to advocate on my behalf for a kidney transplant could never be measured in words. My oncologist placed my unique case on a network of international doctors, many who responded kindly with a positive response to my receiving a living donor's kidney without reserve. Yes, I did receive a living donor's kidney and am extremely thankful to my oncologist, her assistant, nurses, blood lab personnel, those who performed my infusions, physicians who initially diagnosed the issue, the company that provided me with medication and lastly, as a religious individual, the one above.

## Data Availability

All relevant data supporting the findings of this case report are included within the article. No additional datasets were generated or analyzed.
